# Icarust, a real-time simulator for Oxford Nanopore adaptive sampling

**DOI:** 10.1093/bioinformatics/btae141

**Published:** 2024-03-13

**Authors:** Rory Munro, Satrio Wibowo, Alexander Payne, Matthew Loose

**Affiliations:** School of Life Sciences, Medical School, Queens Medical Centre, University of Nottingham, Nottingham NG72RD, United Kingdom; School of Life Sciences, Medical School, Queens Medical Centre, University of Nottingham, Nottingham NG72RD, United Kingdom; School of Life Sciences, Medical School, Queens Medical Centre, University of Nottingham, Nottingham NG72RD, United Kingdom; School of Life Sciences, Medical School, Queens Medical Centre, University of Nottingham, Nottingham NG72RD, United Kingdom

## Abstract

**Motivation:**

Oxford Nanopore Technologies (ONT) sequencers enable real-time generation of sequence data, which allows for concurrent analysis during a run. Adaptive sampling leverages this real-time capability *in extremis*, rejecting or accepting reads for sequencing based on assessment of the sequence from the start of each read. This functionality is provided by ONT’s software, MinKNOW (Oxford Nanopore Technologies). Designing and developing software to take advantage of adaptive sampling can be costly in terms of sequencing consumables, using precious samples and preparing sequencing libraries. MinKNOW addresses this in part by allowing the replay of previously sequenced runs for testing. However, as we show, the sequencing output only partially changes in response to adaptive sampling instructions. Here we present Icarust, a tool enabling more accurate approximations of sequencing runs. Icarust recreates all the required endpoints of MinKNOW to perform adaptive sampling and writes output compatible with current base-callers and analysis pipelines. Icarust serves nanopore signal simulating a MinION or PromethION flow cell experiment from any reference genome using either R9 or R10 pore models. We show that simulating sequencing runs with Icarust provides a realistic testing and development environment for software exploiting the real-time nature of Nanopore sequencing.

**Availability and implementation:**

All code is open source and freely available here—https://github.com/LooseLab/Icarust. Icarust is implemented in Rust, with a docker container also available. The data underlying this article will be shared on reasonable request to the corresponding author.

## 1 Introduction

Nanopore sequencing, as developed by Oxford Nanopore Technologies (ONT), is unique as read data are available for analysis as soon as individual molecules complete passing through the nanopore (Hook and Timp 2023). It is even possible to observe and analyse molecules as they are being sequenced (Loose *et al.* 2016). These properties enable methods such as adaptive sampling whereby individual molecules can be chosen for sequencing from a library (Loose *et al.* 2016, Payne *et al.* 2021). Molecules which are on target are left to finish passing through the pore, whereas off target molecules are rejected from the pore, in a process known as unblocking. This frees up the pore to sequence more molecules. Real-time analysis of sequence data is possible, but tools utilizing these approaches can be complex and costly to develop. Additionally, any tool changing the output of a sequencer could, unintentionally, negatively impact sequencing performance. Therefore thorough testing of tools is required ideally without incurring significant cost.

ONT provides a piece of software, MinKNOW, to control nanopore sequencing. All interactions with the sequencer are conducted via MinKNOW through an open Application Programming Interface (API) (https://github.com/nanoporetech/minknow_api). MinKNOW can be configured to playback prerecorded sequencing data, which enables some simulation options, providing a route to develop tools and analysis methods without significant expense. However, these simulations are limited as reads rejected from a pore are not actually removed from the simulation—instead the original read is fragmented at the point the read would have been unblocked. This results in single original long reads being divided into smaller fragments if they are sent an unblock command. This approach also requires a pre-recorded bulk file for a given sample and experiment (Payne *et al.* 2021). Other simulation tools have been developed to address similar problems. Uncalled, software to implement adaptive sampling functionality, included a simulator that can be driven from pre-existing sequence fast5 files (Kovaka *et al.* 2021). However, to our knowledge this simulator does not remove the need for pre-existing data sets nor can any generic implementation of adaptive sampling be developed to use it. Other ONT signal simulators exist including Squigulator and DeepSignal 1.5 (Li *et al.* 2020, Gamaarachchi *et al.* 2023), although these approaches do not enable real-time simulation for serving squiggle chunks for adaptive sampling.

To address these challenges, we developed Icarust which mimics the functions of MinKNOW. Icarust generates signal data (“squiggle”) derived from the output of “Scrappie” (https://github.com/nanoporetech/scrappie) and serves this signal in real-time using an identical API to the one implemented in MinKNOW itself. Icarust can also generate R10.4 pore squiggle data, and RNA02 squiggle data using models provided by ONT (https://github.com/nanoporetech/kmer_models/). Icarust can simulate any genome, incorporate barcodes and respond to requests to reject reads via the same adaptive sampling API as MinKNOW. Icarust can also simulate amplicon based sequencing experiments. Data from Icarust are written to files (FAST5 or POD5) which are compatible with Oxford Nanopore base-callers. Importantly, this tool is not intended to be used to explore base-calling itself. None of the signal methods employed will capture any of the nuance of real signal, rather they are simply signals that can be meaningfully interpreted by base-callers. This tool enables cheap testing and development of software designed to exploit adaptive sampling and real-time analysis of nanopore sequence data.

## 2 Software implementation

Icarust is implemented in Rust (https://www.rust-lang.org/), using the tonic package (https://github.com/hyperium/tonic/tree/master) to provide gRPC Remote Procedural Call (gRPC) support. Rust was chosen as it was the fastest gRPC implementation (https://github.com/LesnyRumcajs/grpc_bench/wiki/2022-01-11-bench-results), and is naturally asynchronous. An overview of the architecture is shown in Supplementary Fig. S1. All MinKNOW API endpoints required by ReadFish (Payne *et al.* 2021) are implemented.

### 2.1 Squiggle generation

In order to serve signal data (squiggle), we employ one of two methods. For R9 data, we utilize the ONT base-caller Scrappie to convert the reference to an array of squiggle values. These have to be pre-computed as the conversion is slower than sequence data are generated. Icarust then randomly selects a read length and starting location from the pre-computed array for each channel, and serves chunks of this selection, at approximately 4 kHz. This approach is useful as the signal is generated by reversing the base-caller network, but is limited by the large file size of the pre-computed squiggle and the lack of support for later pore types in Scrappie.

The second method utilizes pore models provided by ONT for research purposes (https://github.com/nanoporetech/kmer_models/). We again select a random start point and read length from the reference sequence and convert to signal according to the pore model. The pore models are z-score normalized so must be denormalized for base-calling as in Equation (1).

(1) $$\begin{array}{c} Z-\text{score value}\times \text{Signal Std}.\;\text{dev}+\text{Signal mean} \end{array}$$

Parameter values are chosen sufficient to obtain signal that can be aligned, see the Supplementary repository for notebooks detailing Z-score denormalization. Denormalization is sufficiently fast that is possible to directly convert sequence at the start of the simulation, and so no pre-computation is required. The signal obtained is only sufficient for testing the real-time feedback properties of sequencing, it is not designed to simulate signal perfectly. See Supplementary Fig. S4 for identities of simulated reads.

### 2.2 Barcoding

In order to simulate multiplexed samples, the sequences for the NB12 barcoding kit (ONT) were converted to squiggle. The correct complements are then added to the start and end of the squiggle for a read, and are padded with some “adaptor” signal. The ratio of barcodes in a sample can be altered by providing weights to each barcode in a simulation profile TOML.

### 2.3 Flow cell health modelling

To more accurately recreate the decline of flow cell health across a sequencing run and capture the effects of applying adaptive sampling, we assign each simulated “channel” a probability to become saturated and so unavailable for further sequencing. When simulation begins, some channels are labelled as saturated immediately (default 15%). After a read ends, either naturally or as a result of an unblock, we determine if the channel can produce another read. The probability of this is determined by the base chance (described in Equation (2)) multiplied by the length of the read, divided by 10 000. This takes into account the fact that longer reads are more likely to block a channel than shorter reads.

(2) $$\begin{array}{c} \frac{1.0}{\left(\text{Target yield}/\text{Mean read length}/\text{Number starting channels}\right)} \end{array}$$

These parameters provide a near realistic decay in sequencing performance that mimics the overall total yield of a genuine sequencing experiment. All parameters can be defined by the user (see below).

### 2.4 Configuration

Icarust is configured by a combination of two files. A configuration INI file provides Icarust software specific configuration, such as where TLS certificates can be found for securing the gRPC connection between the MinKNOW API and Icarust, and what ports Icarust listens on. It also provides sequencer specific configuration, such as the number of channels being simulated.

A second configuration TOML file holds the settings about the specific simulation taking place. This “Simulation profile” contains tables that can alter variables about the run, such as the proportions of the species being sequenced, the average read length of the sample, which barcodes are present on each sample. A full list of settings is detailed in the source code README (https://github.com/LooseLab/Icarust/blob/master/README.md).

### 2.5 Output

As with a typical nanopore sequencing run, when 4000 reads have finished sequencing, a FAST5 or POD5 file (by user request) containing the read squiggle and metadata is written out. The directory structure for both the FAST5 and POD5 files mimic the same structure employed by MinKNOW. The availability of these files allows for downstream post run analysis of the simulation.

## 3 Results and discussion

### 3.1 Example use case

**Figure 1.**
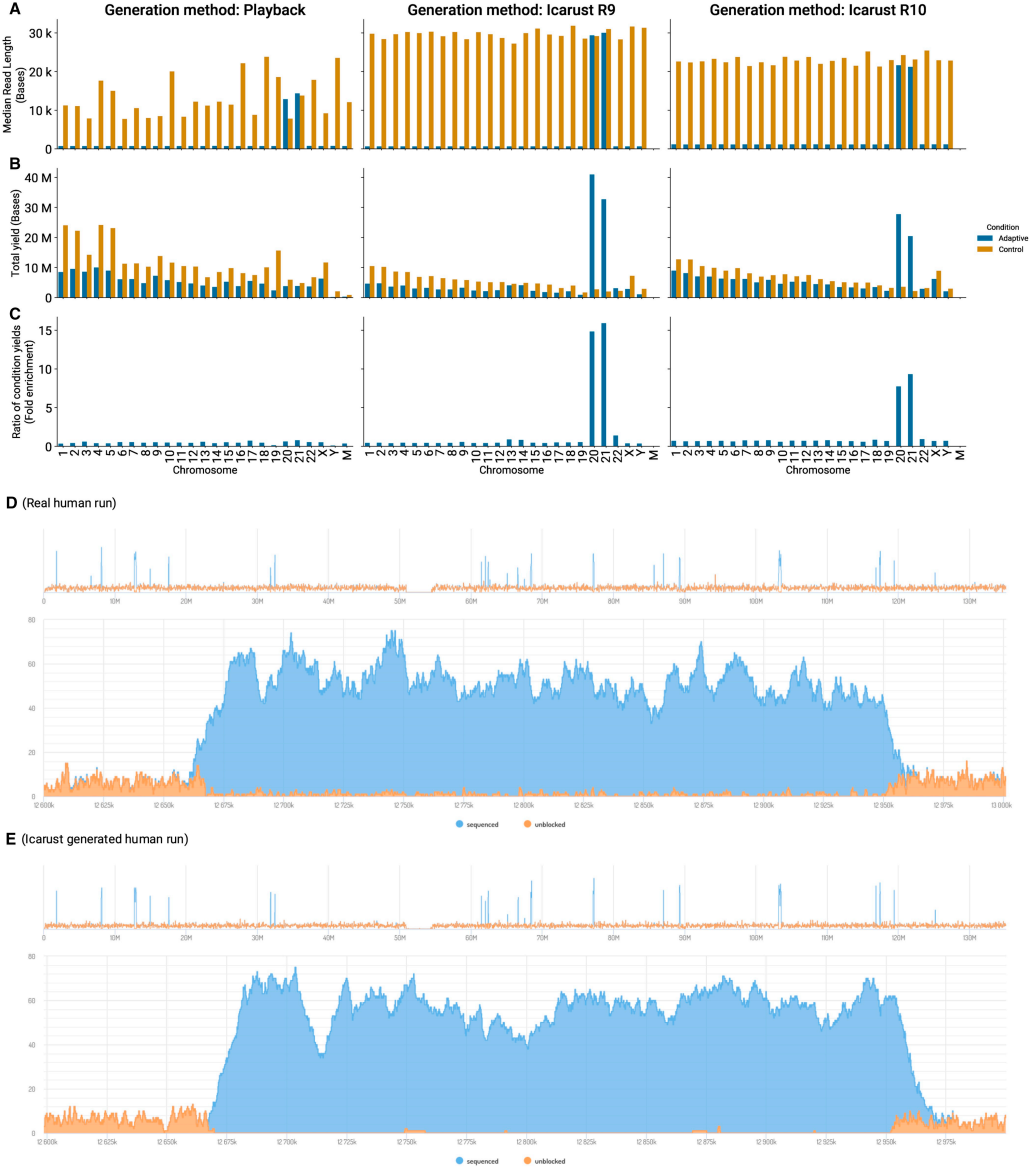
Comparison of an adaptive sampling experiment, targeting Chromosomes 20 and 21 on the human genome, between two simulated runs. The left column visualizes data generated using MinKNOWs playback, the middle column data generated using Icarust with R9 data, the right column data generated using Icarust with R10 data. The two run conditions, Control (orange) or Adaptive sampling (blue) are shown. (A). The median read length of the two runs for each condition. (B). The total yield in bases of the two runs for each condition. (C). The difference between the yield in the Adaptive and Control conditions as the fold change for each run. (D, E) Coverage plots showing the same adaptive sampling target (Chr 11, 12,666,921–12,952,237) from a real human sequencing run, and a replication run, simulated using Icarust R9 data. The top section of the plots shows the whole coverage over Chr 11. Coverage is split between Sequenced (Blue) and Unblocked reads (Orange). Charts plotted by MinoTour.

In order to test the utility of our tool, we compared running adaptive sampling using playback and Icarust with both the R9.4 pore Scrappie model and the R10 model we derived from ONTs pore model values. We performed the simple experiment recommended by ReadFish, targeting Chromosome 20 and 21 on the human genome, on 512 channels (see Supplementary methods). This resulted in 6 runs including controls for playback, Icarust R9 and R10, and then adaptive sampling runs using playback, Icarust R9 and R10. As shown in Fig. 1A, adaptive sampling gave the expected results for each run simulation method, with median read lengths reduced for off target chromosomes. The median read lengths for the on target chromosomes (20 and 21) remained close to, or the same as, the control for both simulation methods.

However the effect adaptive sampling has on the experimental outcome varies by simulation method Fig. 1B and C. The yield (Fig. 1B) for playback runs is lowered when adaptive sampling is applied, likely a consequence of unblocking reads without the ability to replace them with new molecules. As a consequence, there is no enrichment in yield. In contrast, Icarust generated experiments, where reads can be replaced with new molecules, demonstrate clear enrichment of the target chromosomes. Fig. 1C, shows the ratio of the yields between the Control and Adaptive conditions and clearly shows enrichment with Icarust simulations. In the R9 Icarust simulation on target yield is enriched approximately 15× compared to the control, whereas the MinKNOW simulated run has a lower yield around 0.7× the control. In the R10 Icarust simulation, enrichment is similar, if slightly lower. We speculate this reduction is due to the shorter reads in this simulation (20kb compared with 28kb), and an increase in read noise due to our self derived models.

We recreated a human adaptive sampling sequencing run using Icarust, applying the same target set and using ReadFish to perform adaptive sampling. We then uploaded the run to MinoTour (Munro *et al.* 2022) in real-time to monitor enrichment. We could recreate the performance of this run as shown in Fig. 1D and 1E.

## 4 Conclusion

Icarust allows users to quickly and cheaply test adaptive sampling experiments and develop new software for ONT adaptive sampling workflows. Icarust can simulate barcoded (Supplementary Fig. S3) and non-barcoded sequencing runs from any provided reference sequence, sequencing runs using amplicon based libraries (Munro *et al.* 2023), and can simulate both MinION or PromethION scale flow cells with either DNA R9, R10 or RNA02 signal.

Implemented in Rust, Icarust is fast, reliable, memory safe and energy efficient. Icarust is not intended to be a perfect recreation of real squiggle data, instead it is a close enough facsimile to allow software development and testing of experimental setups. The software is freely available, with a maintained docker image allowing easy adoption by the Nanopore Community.

## Supplementary Material

btae141_Supplementary_Data
