# Supplementary material for: Icarust, a real-time simulator for Oxford Nanopore adaptive sampling
Source: Bioinformatics. 2024 Mar 13;40(4):btae141. doi: 10.1093/bioinformatics/btae141 (PMC10980563; doi:10.1093/bioinformatics/btae141)
Supplement: btae141_Supplementary_Data [file btae141_supplementary_data.pdf]

Icarust Supplementary Materials

1 Supplementary figures

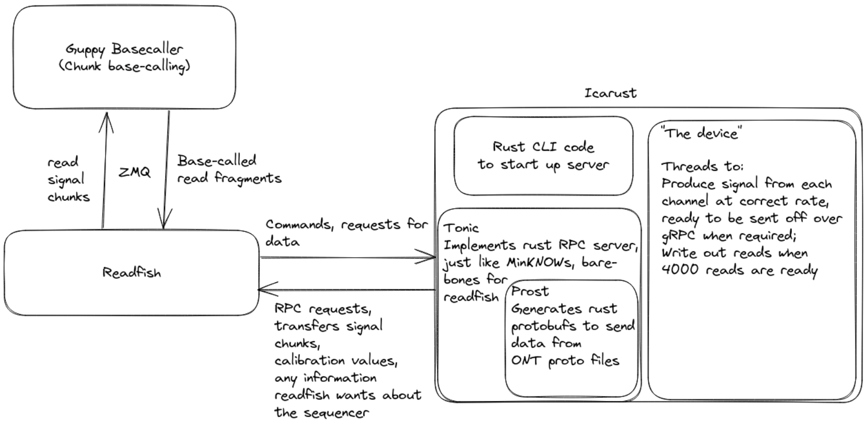

Supplementary Figure 1 – Software architecture of Icarust. The Tonic crate (https://github.com/hyperium/tonic) provides the GRPC implementation, Prost (https://github.com/tokio-rs/prost) compiles the protobufs, and the simulated sequencing device responsible for creating signal data is written in pure rust. ReadFish communicates with Icarust using GRPC calls, in the same structure as ONT's provded MinKNOW API (https://github.com/nanoporetech/minknow_api).

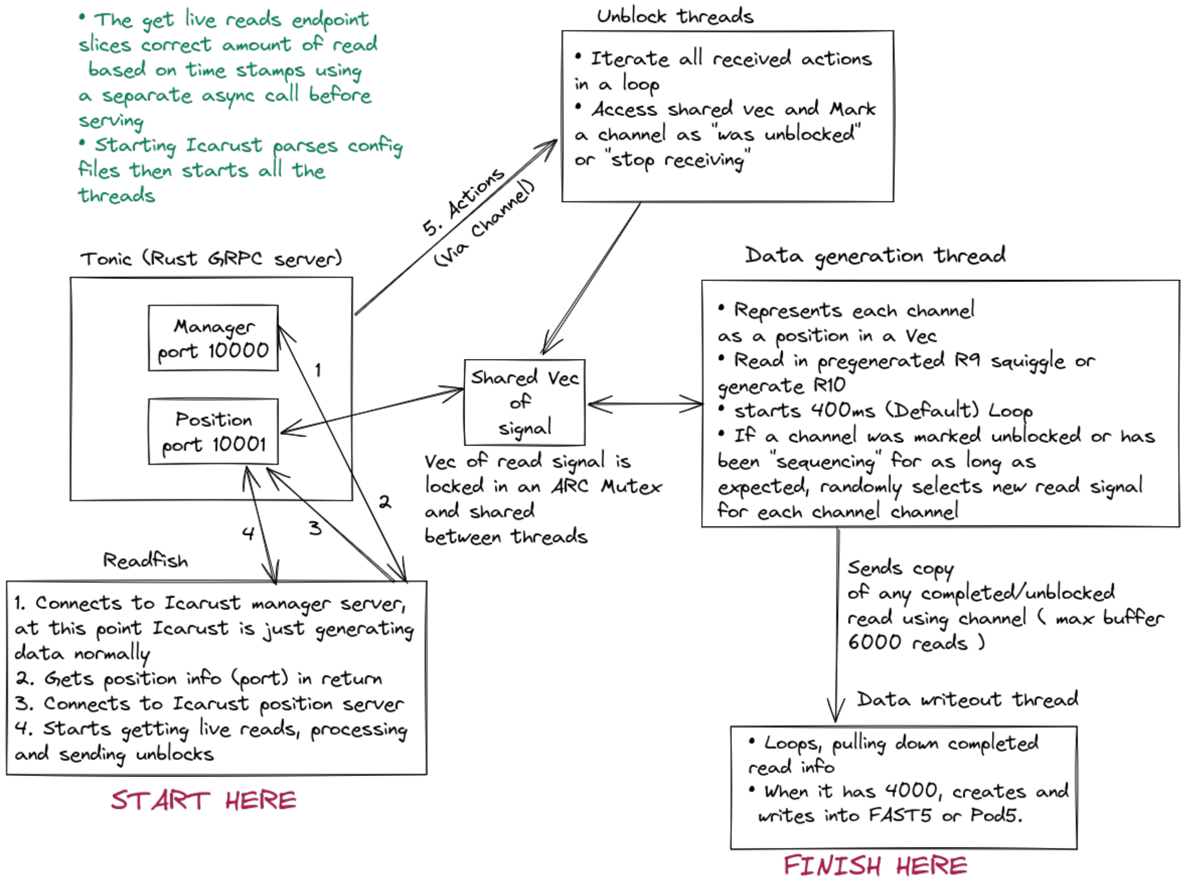

Supplementary Figure 2 – Data flow throughout Icarust. When Icarust launches, it listens for ReadFish connections on port 10000, on a "manager" GRPC server. It also serves a GRPC server "position" on port 10001, which it can serve live data from to readfish. There are three threads, an unblock thread, a data generation thread and a data write out thread. The unblock thread handles any actions that ReadFish has sent via the RPC, either unblock, or stop receiving. The thread labels each channel with the action it has received in the shared vec, which represent all channels. The data generation thread is in charge of selecting signal for each channel, and replacing reads when they would have either been unblocked or trans-located the pore. The data write out thread constantly receives finished reads from the data generation thread over a memory channel, and when it has received 4000, writes them out into a FAST5 or POD5 file. The GRPC server is in a separate asynchronous run time, and access the shared vec of channels to serve signal at the correct rate (roughly 4000 samples a second). This rate is controlled as well by the *break_read_chunks* parameter, serving data at most every *break_read_chunks* ms for a given channel.

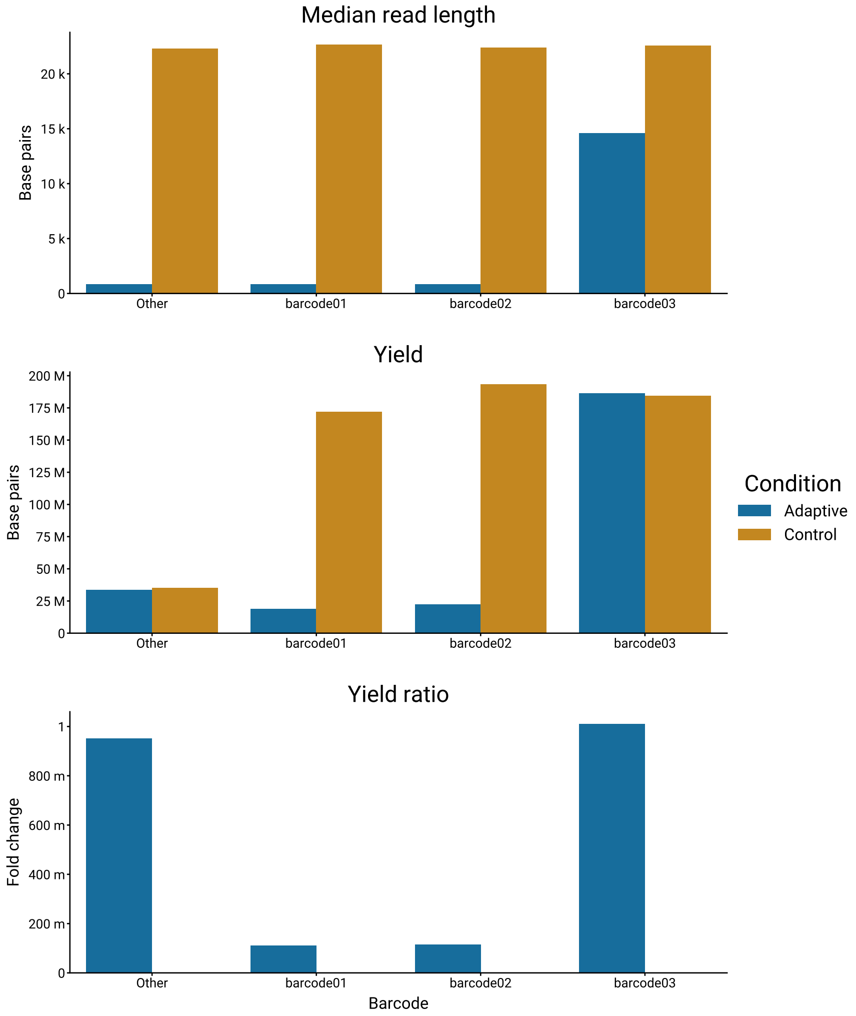

Supplementary Figure 3 – Barcode R10 selection. The control and adaptive experiments were run for an hour each using the same TOML simulation profile in Icarust. Control represents no adaptive sampling applied. Adaptive sampling is configure to reject any read except Barcode03. The upper panel shows median read lengths for each barcode between the two conditions. The middle panel shows the total yield per barcode for each condition. The lower panel shows the yield ratio between control and adaptive conditions.

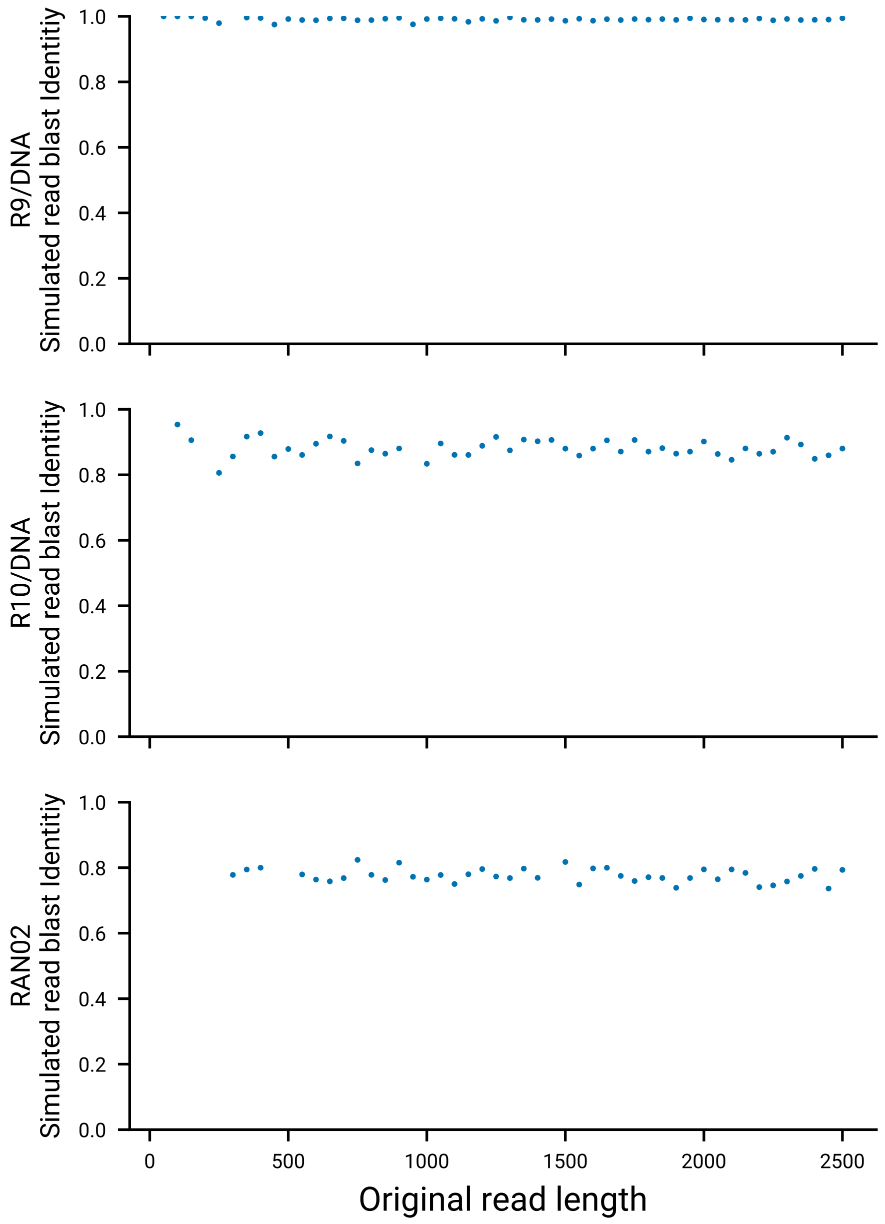

Supplementary Figure 4 – Barcode R10 selection. Alignment Identities of Reads of increasing length for the labelled model types.

2 Supplementary methods

2.1 Chromosome 20 and 21 selection.

Experiments were run for one hour each with and without readfish adaptive sampling enabled. The readfish targets TOML was identical for each run. Experiments were repeated across MinKNOW playback, Icarust R9 and R10 simulation. All basecalling was run using HAC models for the appropriate chemistry. The version of readfish code used can be found at https://github.com/LooseLab/readfish/commit/fb01ea308dde1eeec0a859d3655c15192eacf6ea. The Icarust docker container used was digest a2bd0973c6a2. All simulation and readfish TOMLs are provided in the Supplementary data repository, https://github.com/LooseLab/Icarust_supplementary_data.

2.1.1 MinKNow Playback

MinKNOW playback used a Promethion BETA 48 tower, as described in the ReadFish README.md (https://github.com/LooseLab/readfish#testing).

2.1.2 R9 Icarust simulation

To generate R9 squiggle, hg38 reference seqeunce (RefSeq assembly accession: GCF_000001405.40) was filtered to include the 25 complete chromosomes removing alternative assemblies. Filtering used seqkits' (https://bioinf.shenwei.me/seqkit) grep command. R9 squiggle was then created using icarust as describe in the github README.md.

2.1.3 R10 Icarust simulation

To simulate R10 squiggle, we used the same hg38 reference but with the R10 pore model.

2.2 Barcode selection

A simple barcoded experiment was simulated serving R10 squiggle. The control (no adaptive sampling) and adaptive (adaptive sampling) conditions were run for an hour each. The experiment was setup to serve barcodes 01, 02 and 03 with equal probability. In the adaptive condition, any read which was not demultiplexed to barcode03 was rejected, as shown in Supplementary fig. 4. The reference served contained two bacterial sequences, *Bacillus anthracis* str. Ames and *Pseudomonas aeruginosa* PA01. This reference file can be found in the supplementary data repository, under experimental files.
